# DRIM: A Web-Based System for Investigating Drug Response at the Molecular Level by Condition-Specific Multi-Omics Data Integration

**DOI:** 10.3389/fgene.2020.564792

**Published:** 2020-11-12

**Authors:** Minsik Oh, Sungjoon Park, Sangseon Lee, Dohoon Lee, Sangsoo Lim, Dabin Jeong, Kyuri Jo, Inuk Jung, Sun Kim

**Affiliations:** ^1^Department of Computer Science and Engineering, Seoul National University, Seoul, South Korea; ^2^Bioinformatics Institute, Seoul National University, Seoul, South Korea; ^3^Interdisciplinary Program in Bioinformatics, Seoul National University, Seoul, South Korea; ^4^Department of Computer Engineering, Chungbuk National University, Cheongju, South Korea; ^5^Department of Computer Science and Engineering, Kyungpook National University, Daegu, South Korea; ^6^Department of Computer Science and Engineering, Institute of Engineering Research, Seoul National University, Seoul, South Korea

**Keywords:** multi-omics, drug-response, time-series, perturbed pathway, web-system, pharmacogenomics

## Abstract

Pharmacogenomics is the study of how genes affect a person's response to drugs. Thus, understanding the effect of drug at the molecular level can be helpful in both drug discovery and personalized medicine. Over the years, transcriptome data upon drug treatment has been collected and several databases compiled before drug treatment cancer cell multi-omics data with drug sensitivity (*IC*_50_, AUC) or time-series transcriptomic data after drug treatment. However, analyzing transcriptome data upon drug treatment is challenging since more than 20,000 genes interact in complex ways. In addition, due to the difficulty of both time-series analysis and multi-omics integration, current methods can hardly perform analysis of databases with different data characteristics. One effective way is to interpret transcriptome data in terms of well-characterized biological pathways. Another way is to leverage state-of-the-art methods for multi-omics data integration. In this paper, we developed Drug Response analysis Integrating Multi-omics and time-series data (DRIM), an integrative multi-omics and time-series data analysis framework that identifies perturbed sub-pathways and regulation mechanisms upon drug treatment. The system takes drug name and cell line identification numbers or user's drug control/treat time-series gene expression data as input. Then, analysis of multi-omics data upon drug treatment is performed in two perspectives. For the multi-omics perspective analysis, *IC*_50_-related multi-omics potential mediator genes are determined by embedding multi-omics data to gene-centric vector space using a tensor decomposition method and an autoencoder deep learning model. Then, perturbed pathway analysis of potential mediator genes is performed. For the time-series perspective analysis, time-varying perturbed sub-pathways upon drug treatment are constructed. Additionally, a network involving transcription factors (TFs), multi-omics potential mediator genes, and perturbed sub-pathways is constructed, and paths to perturbed pathways from TFs are determined by an influence maximization method. To demonstrate the utility of our system, we provide analysis results of sub-pathway regulatory mechanisms in breast cancer cell lines of different drug sensitivity. DRIM is available at: http://biohealth.snu.ac.kr/software/DRIM/.

## 1. Introduction

The variability in drug responses among cells is a major challenge in cancer drug therapy, thus personalized drug response research is much needed (Sweeney, 1983). With the recent advances in instrument technologies, drug response analysis at the molecular level has become possible, thus we have an opportunity to investigate relationship between drug response phenotypes and corresponding molecular data, for example, multi-omics data upon drug treatment. Large-scale drug response genomics data help identify molecular markers related with therapeutic response (Garnett et al., 2012). Furthermore, more than 100 US Food and Drug Administration (FDA)-approved drugs have been developed from rapidly growing pharmacogenomics studies. This shows that pharmacogenomics data could be used for drug development at various stages, from drug targets to patient therapeutics. Moreover, genomics data of the patient can be regarded as a predictive factor for drug response. It can be thought of as an early response signal before the phenotypic change of cells by drug (Surendiran et al., 2008).

Current pharmacogenomics data analysis can be extended in two directions to broaden the understanding of drug response. The first direction is to perform a pathway-level analysis. Analyzing drug responses at the individual gene level is difficult to explain biological variability and also difficult to interpret gene-drug associations (Wang et al., 2019). Thus, focus of pharmacogenomics research is changing to investigate multiple gene products at the biological pathway level (Weinshilboum and Wang, 2004). A recent study shows that analysis of transcriptome data can be effectively done at the pathway level, which facilitates clear biological interpretation (Lim et al., 2020). The second direction is to perform multi-omics level analysis. Recently, precision medicine studies have been conducted at the multi-omics level, which is called “pharmaco-omics” beyond pharmacogenomics by integrating genomics, proteomics, epigenomics, and metabolomics data (Adam and Aliferis, 2019; Ginsburg et al., 2019). Many studies have shown that multi-omics integration helps unravel complex biological mechanisms (Subramanian et al., 2020). Integrative analysis of multi-omics data can help understand cell line-specific gene regulation mechanisms for pathway activation (Kim et al., 2016; Oh et al., 2020) and it can be used as a signature for drug response sub-pathway identification (Xu et al., 2019). Single omics analysis can detect only a smaller subset, but multi-omics analysis can detect more comprehensive pathways that respond to chemical exposure (Canzler et al., 2020).

There are several pharmacogenomics databases such as Genomics of Drug Sensitivity in Cancer (GDSC) (Iorio et al., 2016), Cancer Cell Line Encyclopedia (CCLE) (Barretina et al., 2012), Patient-Derived Xenograft (PDX) mice models (Gao et al., 2015), and NCI-60 Human Tumor Cell Lines Screen (Abaan et al., 2013). These databases can be used for cell line-specific drug sensitivity analysis with multi-omics signature at the molecular level. In addition, data from after drug treatment time-series experiments can be used to capture time-varying cell line-specific drug response as signature of cell death, proliferation, and drug resistance. The Library of Integrated Network-based Cellular Signatures (LINCS) L-1000 (Subramanian et al., 2017) project measures cell viability upon genetic and chemical perturbations by 978 landmark genes. Another database compiled time-series transcriptome data using the NCI-60 cell line upon anti-cancer drug treatment (Monks et al., 2018).

There are several databases that enable computational pharmacogenomics study. GDSC measured the response of 988 cell lines to 518 drug compounds (Iorio et al., 2016). It provides mutation, copy number variation, DNA methylation, and gene expression data of cell lines before drug treatment. CCLE (Barretina et al., 2012) measured genomics profiles and response to 24 anticancer drugs in 947 cell lines. A recent study (Ghandi et al., 2019) performed RNA sequencing (RNA-seq), whole-exome sequencing (WES), whole-genome sequencing (WGS), reverse-phase protein array (RPPA), reduced representation bisulfite sequencing (RRBS), microRNA expression profiling, histone modification profiling, metabolites profiling (Li et al., 2019), and 1,448 drugs response (Corsello et al., 2020) for CCLE cell lines. NCI-60 cell lines are the most widely studied cell lines in human cancer research. CellMiner (Reinhold et al., 2012) is a website that provides 20,503 chemical compounds response of NCI-60 cells and also genomics data before drug treatment as mutation, DNA methylation, microRNA expression, gene expression, and protein data. The NCI Transcriptional Pharmacodynamics Workbench (NCI TPW) (Monks et al., 2018) provides time-series pharmacogenomics data and a web page that allows data exploration. They measured that gene expression changes the NCI-60 cell line after drug exposure of 2, 6, and 24 h to 15 anticancer drugs. NCI-DREAM community (Bansal et al., 2014) measured that gene expression changes the OCI-LY3 cell line after 14 anticancer drug treatment for 6, 12, and 24 h to predict the activity of pairs of compounds.

By utilizing pharmacogenomics data in various databases, a number of studies have been performed to analyze pharmacogenomics data in terms of IC50 prediction and drug response gene/pathway identification. Table 1 summarizes pharmacogenomics data analysis methods. Multi-Omics Late Integration (MOLI) (Sharifi-Noghabi et al., 2019) is an end-to-end deep neural network-based drug response prediction method. MOLI takes mutation, copy number, and gene expression as input, and predicts drug response using each omics type-specific encoder. Drug Sensitivity Prediction using a novel regularization approach in Logistic Matrix Factorization (DSPLMF) (Emdadi and Eslahchi, 2020) is a drug response prediction method based on recommender systems. DSPLMF takes cell line similarity matrix consisted of gene, copy number, mutation, and IC50 and drug similarity matrix as input, and predicts drug response using matrix factorization and nearest neighbor algorithm. CancerDAP (Xu et al., 2019) is a pipeline that integrates gene expression, copy number variation, and DNA methylation to identify sub-pathway signature of anticancer drug response. The user can browse drug active sub-pathway using CancerDAP web page. Differential regulatory Network-based Modeling and Characterization (DryNetMC) (Zhang et al., 2019) is a network-based algorithm to detect key cancer resistance genes based on time-series RNA-seq data. DryNetMC uses time-series RNA-seq data after drug treatment as input. From the data, it constructs drug-sensitive network and drug-resistant network utilizing ordinary differential equations and extracts differential network. Using differential network, a node importance is measured by topology, entropy, and gene expression changes to prioritize genes of clinical relevance.

**Table 1:** Pharmacogenomics data analysis methods, their input, output, and algorithms.

**Method**	**Input**	**Output**	**Algorithm**
MOLI	Multi-omics data	Drug response (IC50)	Deep learning
DSPLMF	Multi-omics data, chemical structures	Drug response (IC50)	Logistic Matrix Factorization
CancerDAP	Multi-omics data	Sub-pathway signatures for drug response	Random forest, logistic regression
DryNetMC	Drug treatment time-series gene expression data	Clinically relevant genes	Differential network analysis

Lv et al. (2018) performed an analysis of differentially expressed genes (DEGs) on hepatocellular carcinoma (HCC) patients for drug discovery from gene expression data. They divided HCC patients into two groups: high/low-PKM2 to investigate the effect of pyruvate kinase isozymes M2 (PKM2) gene expression on HCC patients in terms of metabolic changes and prognosis. The study identified metabolic genes related to poor HCC patient survival and screened drugs that target metabolic enzymes associated with poor survival. Some of the screened drugs have been used in antitumor clinical studies. Another study proposed a tensor decomposition-based drug discovery method for neurological disorder from gene expression data (Taguchi and Turki, 2019). They selected genes through tensor decomposition-based feature extraction using mouse Alzheimer's single-cell RNA-seq data. These genes are significantly overlapped with the target genes of Alzheimer's disease drugs. Recently, a deep learning-based generative model (Méndez-Lucio et al., 2020) proposed to design active-like molecules from gene expression signatures. The generative model takes the desired gene expression profile induced by drug treatment or gene knock-out experiment as input. The study generates a molecular representation that is likely to have caused a change in gene expression.

## 2. Motivation

To utilize rapidly accumulating drug response omics data, many computational methods for drug response prediction have been developed. Machine learning methods are often used to process high-dimensional genomics data, such as matrix-factorization models (Brouwer and Lió, 2017; Wang et al., 2017), network-based models (Zhang et al., 2015, 2018), and deep learning models (Sharifi-Noghabi et al., 2019; Baptista et al., 2020). Moreover, analysis methods for time-series omics data have been developed (Jo et al., 2016; Ahn et al., 2019; Kang et al., 2019; Kim et al., 2019). However, utilizing these tools for the analysis of pharmacogenomics databases requires expert-level bioinformatics skill.

**Figure 1:**
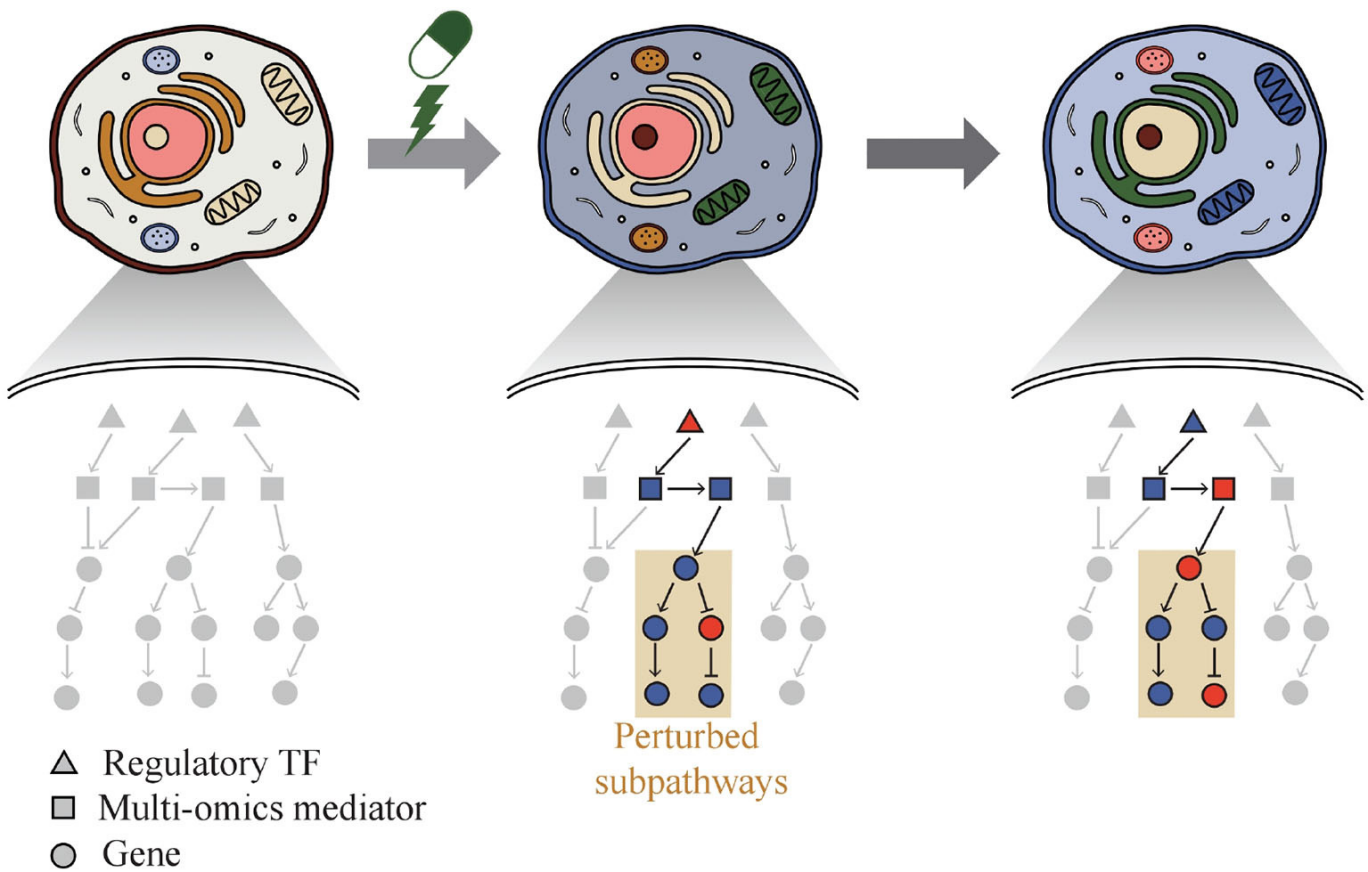
Phenotypic change of cell over time by drug. DRIM makes it possible to interpret drug response at molecular level by investigating perturbed sub-pathways.

Thus, a web-based system called Drug Response analysis Integrating Multi-omics and time-series data (DRIM) was developed and presented in this paper by integrating condition-specific multi-omics data to investigate temporal drug response at the molecular level like Figure 1. The condition of the sample can be defined as a combination of three variables that are cell line type, drug type, and drug dose. DRIM aims to identify perturbed sub-pathways and regulatory mechanisms upon drug treatment using an integrative analysis framework on both multi-omics and time-series data. By simply taking drug name and cell line ID or user's drug control/treat time-series gene expression data as input, DRIM performs the analysis in two perspectives. First, *IC*_50_-related multi-omics potential mediator genes are chosen by embedding multi-omics data into gene-centric vector space using either a tensor decomposition or an autoencoder deep learning model. The tensor decomposition does not require pre-training to determine relationship among different omics components. Feature space from tensor decomposition is linear combination of input features, thus it is easy to interpret how the feature space combines input features. On the other hand, the autoencoder can learn nonlinear relationship of multi-omics data. Autoencoder requires pre-training but it can generate a feature space dynamically for new incoming multi-omics data. In terms of computation time, tensor decomposition is faster than the autoencoder. Then, the potential mediator genes are extended to the identification of perturbed pathways upon drug treatment over time. This time-series analysis construct a network containing transcription factors (TFs), multi-omics mediator genes, and perturbed sub-pathways by an influence maximization-based method.

To demonstrate the utility of our system, we provide analysis results of sub-pathway regulatory mechanisms in breast cancer cell lines of different breast cancer drug sensitivity.

## 3. Methods

**Figure 2:**
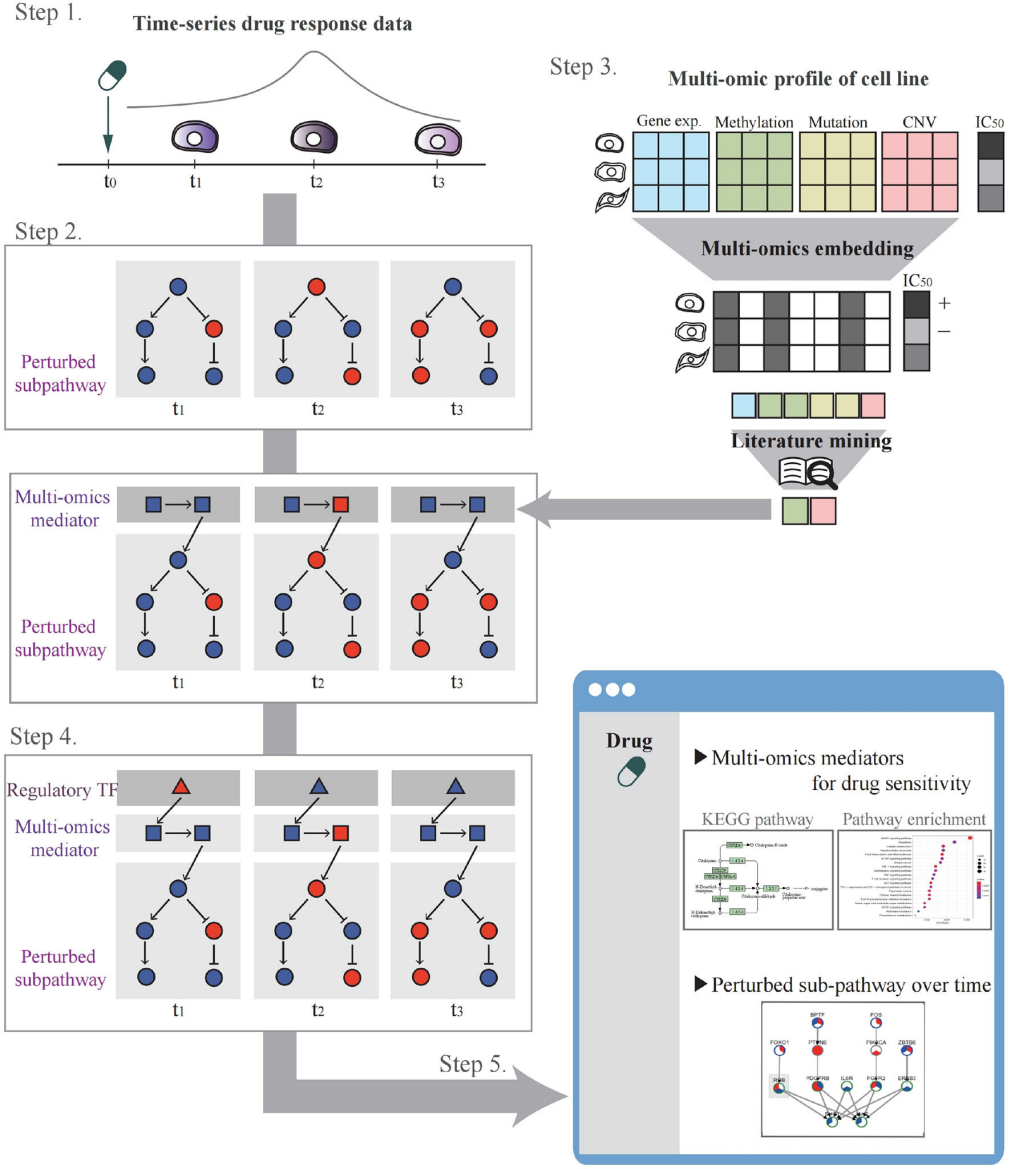
The systematic workflow of the system. Step 1 is for drug and cell line selection. Step 2 is for perturbed sub-pathway identification using expression propagation. Step 3 is for selecting multi-omics potential mediator genes by multi-omics embedding methods. Step 4 is for constructing time-bound network and determining regulatory path by influence maximization. Step 5 is to visualize the analysis result.

The system workflow is illustrated in Figure 2. In Step 1, the user selects a drug and cell lines to be analyzed for perturbed pathway analysis or uploads their drug control/treat time-series gene expression data. In Step 2, through time-series gene expression data analysis after drug treatment, perturbed sub-pathways are identified. In Step 3, multi-omics potential mediator genes are selected by multi-omics integration methods. In Step 4, a time-bounded network is constructed and the most regulatory path is identified by influence maximization. In Step 5, the system visualizes networks involving TF, mediator genes, and perturbed sub-pathways that change over time upon drug treatment. A detailed description of each step in the workflow is as follows.

### 3.1. Step 1: Input

The user selects a drug and cell lines to be analyzed for perturbed pathway analysis or uploads their own drug control/treat time-series gene expression data. The system uses two time-series gene expression after drug treatment databases LINCS L-1000 and NCI-60. In both databases, there are control and treated data for drugs per cell line. For each condition, the gene expression was measured at each time point. These databases are available as GSE70138 and GSE116438 in GEO.

### 3.2. Step 2: Identifying Perturbed Sub-pathway With Time Series

Step 2 is for identifying perturbed sub-pathways of DEGs that are defined using a time-series data analysis tool, TimeTP (Jo et al., 2016). First, each pathway is represented as a directed graph from the KEGG pathway database. For each node in the pathway, the system assigns a time vector $\vec{v}$ of 1 (overexpressed) or −1 (underexpressed) and 0 (unchanged) that are defined by comparing gene expression levels, treated vs. control. Limma (Smyth, 2005; Ritchie et al., 2015) was used to define DEGs at each time point with robust multiarray average (RMA) normalization (Kupfer et al., 2012). When there is no control sample, differential expression genes are defined by comparing either to the expression level of the previous time point or to the expression level of initial time point. Second, a perturbed sub-pathway is determined by choosing valid edges in the pathway graph. Assume that there is an edge *N*1 → *N*2 between two genes, *N*1 and *N*2, that have differential time vectors $\vec{v_{1}}$ and $\vec{v_{2}}$. To measure the direction of propagation and the number of delayed time points between two vectors, cross-correlation is defined as

(1) $$\begin{array}{l} \left(\vec{v_{1}}⋆\vec{v_{2}}\right)\left(n\right)=\overset{\infty }{\underset{t=-\infty }{\sum }}\vec{v_{1}}\left(t\right)\vec{v_{2}}\left(t+n\right) \end{array}$$

where $\vec{v}\left(t\right)=0$ for *t* ≤ 0 or *t* > *T* (this happens at the preceding or trailing entries of two vectors). Cross-correlation is maximized when the two vectors overlap most with *n* delay.

(2) $$\begin{array}{l} d\left(\vec{v_{1}},\vec{v_{2}}\right)=\underset{n}{\mathrm{argmax}}\left(\vec{v_{1}}⋆\vec{v_{2}}\right)\left(n\right) \end{array}$$

If $d\left(\vec{v_{1}},\vec{v_{2}}\right)$ is negative, it means that the propagation direction is opposite to the given direction. The opposite edge is considered as invalid and excluded from the perturbed sub-pathway. When delay *n* is larger than a threshold value, the edge is filtered out. After choosing valid edges, a sub-graph that has more than two valid edges is determined as a perturbed sub-pathway. *P*-value of a perturbed sub-pathway is determined by permutation test. The null distribution is generated by randomly re-assigning differential expression vector for each gene in the sub-pathways. A sum of cross-correlations of edges is used as a pathway-level statistics and *P*-value for a perturbed sub-pathway is calculated from the null distribution.

### 3.3. Step 3: Embedding Multi-Omics for Selecting Potential Mediator Genes

Step 3 determines potential mediator genes related to drug sensitivity from the multi-omics regulation perspective. The system integrates four multi-omics data such as gene expression, copy number variation, DNA methylation, and mutation from the CCLE database. Each omics data processed to a gene-centric (*cellline* × *gene*) matrix to discover potential mediator genes from the perspective of multi-omics regulation. The gene expression and copy number variation values were normalized by min–max normalization. The mutation data were binarized to 1 if mutations exist in the gene or 0 otherwise. To process methylation data, methylation levels of probes located within the transcription start site and 1 KB upstream of promoter regions were averaged per gene. The *IC*_50_ value measured for each cell line is used as the drug response phenotype.

The system uses two machine learning algorithms, a tensor-decomposition method and an autoencoder method, to embed high-dimensional multi-omics data to low-dimensional feature space. The embedding of the multi-omics data is to create a “gene-centric” feature space, which means that regulation information, such as copy number variation, DNA methylation, and mutation, is tied to a gene while embedding multi-omics data.

**Figure 3:**
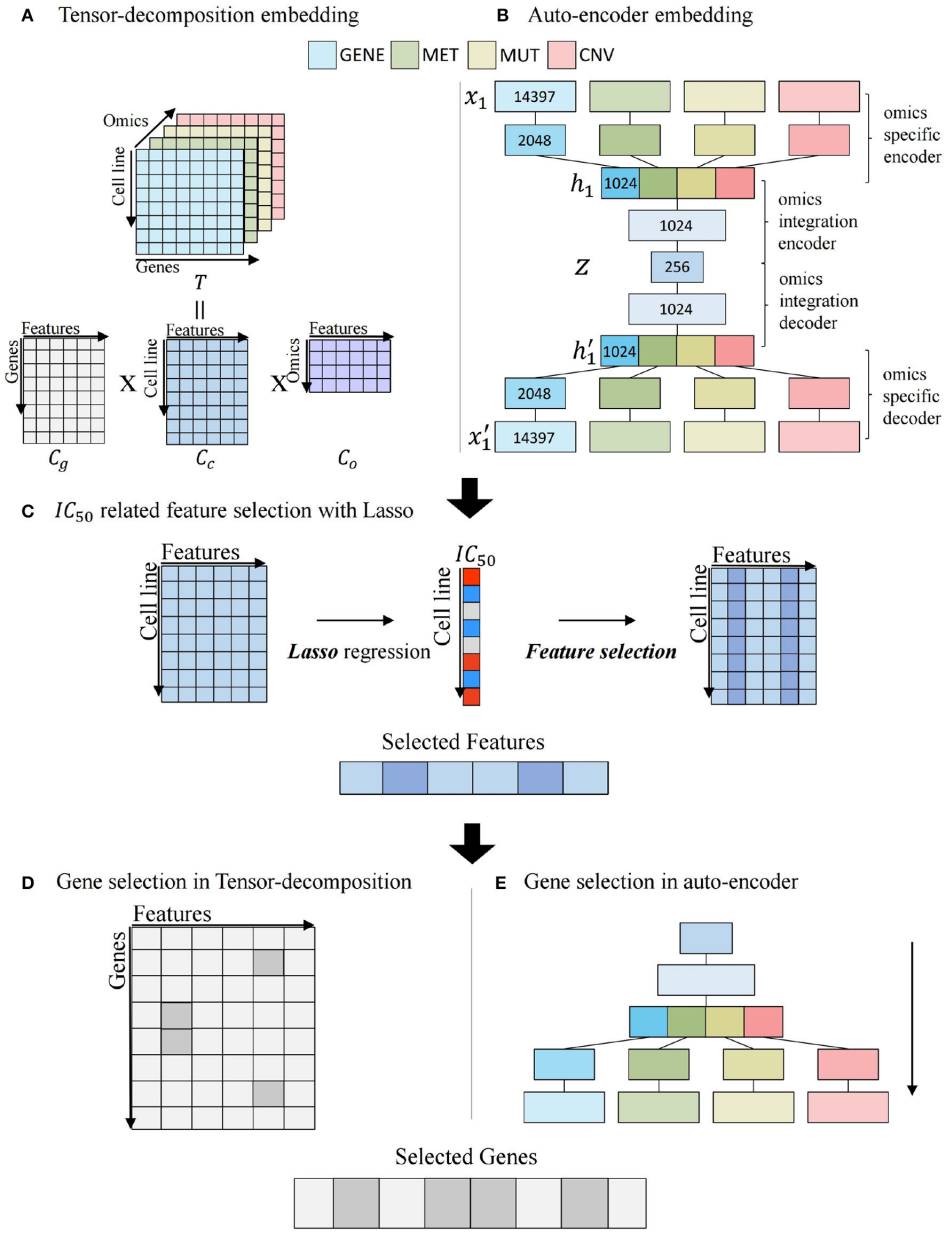
Multi-omics potential mediator gene selection. **(A)** Multi-omics integration by tensor decomposition. **(B)** Multi-omics integration by autoencoder. **(C)** *IC*_50_-related feature selection using Lasso regression with embedded feature matrix. **(D)** Gene selection of tensor decomposition from selected features. **(E)** Gene selection of autoencoder from selected features.

Figures 3A,B illustrates the process of embedding gene-centric multi-omics data with two algorithms. For tensor decomposition, we used the PARAFAC model that decomposes a tensor into three two-dimensional matrices (Rabanser et al., 2017). As shown in Figure 3A, tensor *T* with elements *x*_*ijk*_ composed of *cell line* × *gene* × *omics* matrix and is factorized into three matrix *C*_*g*_, *C*_*c*_, and *C*_*o*_ with *g*_*if*_, *c*_*jf*_, and *o*_*kf*_. *C*_*g*_, *C*_*c*_, and *C*_*o*_ are defined as gene, cell line, and omics components, respectively. *f* = 1, ...., *R*, *R* is the number of features.

(3) $$\begin{array}{l} x_{ijk}=\overset{R}{\underset{f=1}{\sum }}g_{if}c_{jf}o_{kf}+e_{ijk} \end{array}$$

We used *C*_*c*_ matrix that embeds cell line–specific multi-omics relationship.

Figure 3B describes the process of autoencoder embedding that is unsupervised artificial neural network to learn efficient encoded representation of data (Kramer, 1991). We constructed a late-integration autoencoder that encodes gene-centric multi-omics data. An input vector is represented as *x* = (*x*_1_, ..., *x*_*n*_, *x*_*n*+1_, ..., *x*_2*n*_, *x*_2*n*+1_, ..., *x*_3*n*_, *x*_3*n*+1_, ..., *x*_4*n*_) that is a concatenation of four multi-omics values and *n* is the number of genes. An autoencoder is to reconstruct *x*′ as output for an input vector *x*. For each layer *l*, we used *relu* as activation function between input layer *x* and output layer *y*.

(4) $$\begin{array}{l} y=f_{l}\left(x\right)=relu\left(W_{l}x+b_{l}\right) \end{array}$$

The autoencoder consists of four system components: an omics-specific encoder, an omics-integration encoder, an omics-integration decoder, and an omics-specific decoder. In the omics-specific encoder, features are learned individually for each omics data. For each omics data of *x*_*i*_ with *i* = (1, 2, 3, 4), *x*_*i*_ is encoded to *h*_*i*_.

(5) $$\begin{array}{l} h_{i}=F^{k}\left(x\right)=f_{k}\circ ...\circ f_{1}\left(x\right) \end{array}$$

where *k* is the number of layer, and *f*_*k*_ ∘ *f*_*k*−1_(*x*) = *f*_*k*_(*f*_*k*−1_(*x*)) is the composition function of *f*. The omics-integration encoder learns relationship among multi-omics data using concatenated omics features *h* = (*h*_1_, *h*_2_, *h*_3_, *h*_4_) and encodes *h* to *z* in a similar way to Equation (5). *z* is an embedding vector that learns the regulation of multi-omics relationship. The omics-integration decoder decodes *z* to *h*′. The omics-specific decoder decodes omics specific $h_{i}^{\prime }$ to $x_{i}^{\prime }$ and reconstruct input $x^{\prime }=\left(x_{1}^{\prime },x_{2}^{\prime },x_{3}^{\prime },x_{4}^{\prime }\right)$ in the opposite way to the encoder. For each encoder and decoder, we used 2 layers and 2,048, 1,024 hidden neurons in the omics specific layers, and 1,024, 256 hidden neurons in the omics integration layers. We used *mean squared error* (*MSE*) *loss* as a loss function with *L*2 regularization on the weight vector such as Equation (6).

(6) $$\begin{array}{l} Loss=\overset{N}{\underset{i=1}{\sum }}\frac{1}{N}{\left(x_{i}-x_{i}^{\prime }\right)}^{2}+\lambda *\overset{P}{\underset{i=1}{\sum }}|w_{i}| \end{array}$$

*N* is the number of data, *P* is the number of layer, and *w*_*i*_ is the weight of *i*th layer. Figure 3C illustrates the feature selection process, using *C*_*c*_ matrix by tensor decomposition or *z* vector by autoencoder multi-omics embedding matrix. Least Absolute Shrinkage and Selection Operator (LASSO) regression model (Tibshirani, 1996) is constructed using *IC*_50_ as a target value. Features with non-zero coefficients in regression are considered as features that are significantly associated with the *IC*_50_ value.

Figures 3D,E depicts the gene selection step related to associated features from Figure 3C. In Figure 3D, tensor-decomposition method using *C*_*g*_ matrix is for gene selection. For each gene, the row-wise *argmax* operation can be used to obtain the feature most related to the gene, and if the feature is among the *IC*_50_-related features obtained in the previous step (features whose coefficients are large in Lasso regression), the gene is selected. The product of *C*_*g*_(*g, f*) and *coef*(*f*) is defined as the omics score of the gene, where *coef*(*f*) is the coefficient of *f*′*th* feature in Lasso regression.

The autoencoder method uses decoder part for gene selection in Figure 3E. To evaluate features of a gene in terms of multi-omics, a process-selected feature in the decoder is activated and propagated to the omics data layer. Activation of the final layer is measured through the gene-wise summation and the omics score is computed. The significant genes related with the features are selected.

*Selection of multi-omics potential mediator genes* is done by combining the two scores, a condition-specific omics score and a literature-based score using BEST, a biomedical entity search tool (Lee et al., 2016). When a drug name is submitted to the BEST system, genes that are known to be related to the drug are selected in a ranked list in the order of relevance to the drug. Combining the two scores is done by a method that was developed for microRNA and target gene interaction (Oh et al., 2017).

### 3.4. Step 4: Construct TF-Regulatory Time-Bounded Network and Identify Regulatory Path

Step 4 is for constructing TF-regulatory time-bounded network and determining regulatory paths. First, two networks are constructed to search upstream regulators of perturbed sub-pathway. A gene regulatory network (GRN) is constructed from HTRIdb (Bovolenta et al., 2012) for interaction information between TF and multi-omics potential mediator. A protein-interaction network (PIN) is instantiated from STRING (Szklarczyk et al., 2015) database for gene-gene interaction. To combine GRN, PIN, and perturbed sub-pathways as TF-regulatory time-bounded networks, we used the method described in Step 2.

Next, the most likely regulatory paths are identified by the influence maximization method that has been widely used to select marketing targets in the social network to maximize the spread of influence (Kempe et al., 2003). Our system uses a labeled influence maximization algorithm (Li, 2011) to the time-bounded network to identify most influential regulatory path from TF to perturbed sub-pathway (Jo et al., 2016).

### 3.5. Step 5: Analysis Result on the Web

The system provides analysis results on the web from two perspectives: multi-omics data before drug treatment and time-series gene expression data after drug treatment.

#### 3.5.1. Multi-Omics Analysis Result Before Drug Treatment

**Figure 4:**
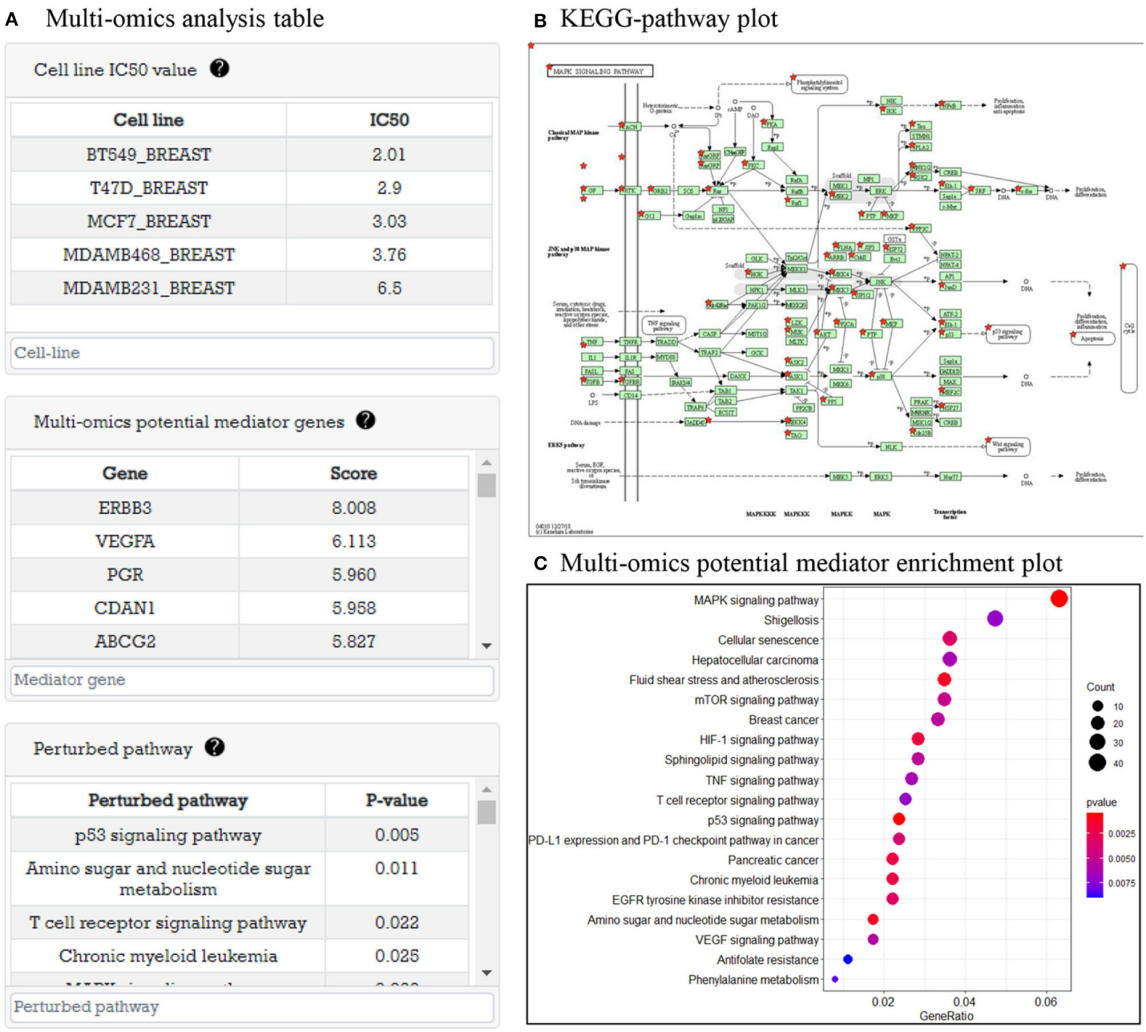
Multi-omics data analysis result before drug treatment. **(A)** Three tables are shown: cell line with *IC*_50_ table, multi-omics potential mediator genes with score table, and perturbed pathway with *P*-value table. **(B)** Perturbed pathway mapping to KEGG pathway. **(C)** An enriched pathway dot plot.

In this part, system provides analysis results of multi-omics data before drug treatment. As an example, in Figure 4A, there are tables representing cell line *IC*_50_, multi-omics potential mediator genes related to *IC*_50_ value, and perturbed pathways that are enriched by potential mediator genes. In Figure 4B, when the user clicks on the pathway in the pathway table, a KEGG pathway plot is created. Figure 4C is GO enrichment analysis plot of potential mediator genes to show the biological functions of the multi-omics potential mediator gene set in relation to drug sensitivity.

#### 3.5.2. Time-Series Gene Expression Analysis Result After Drug Treatment

**Figure 5:**
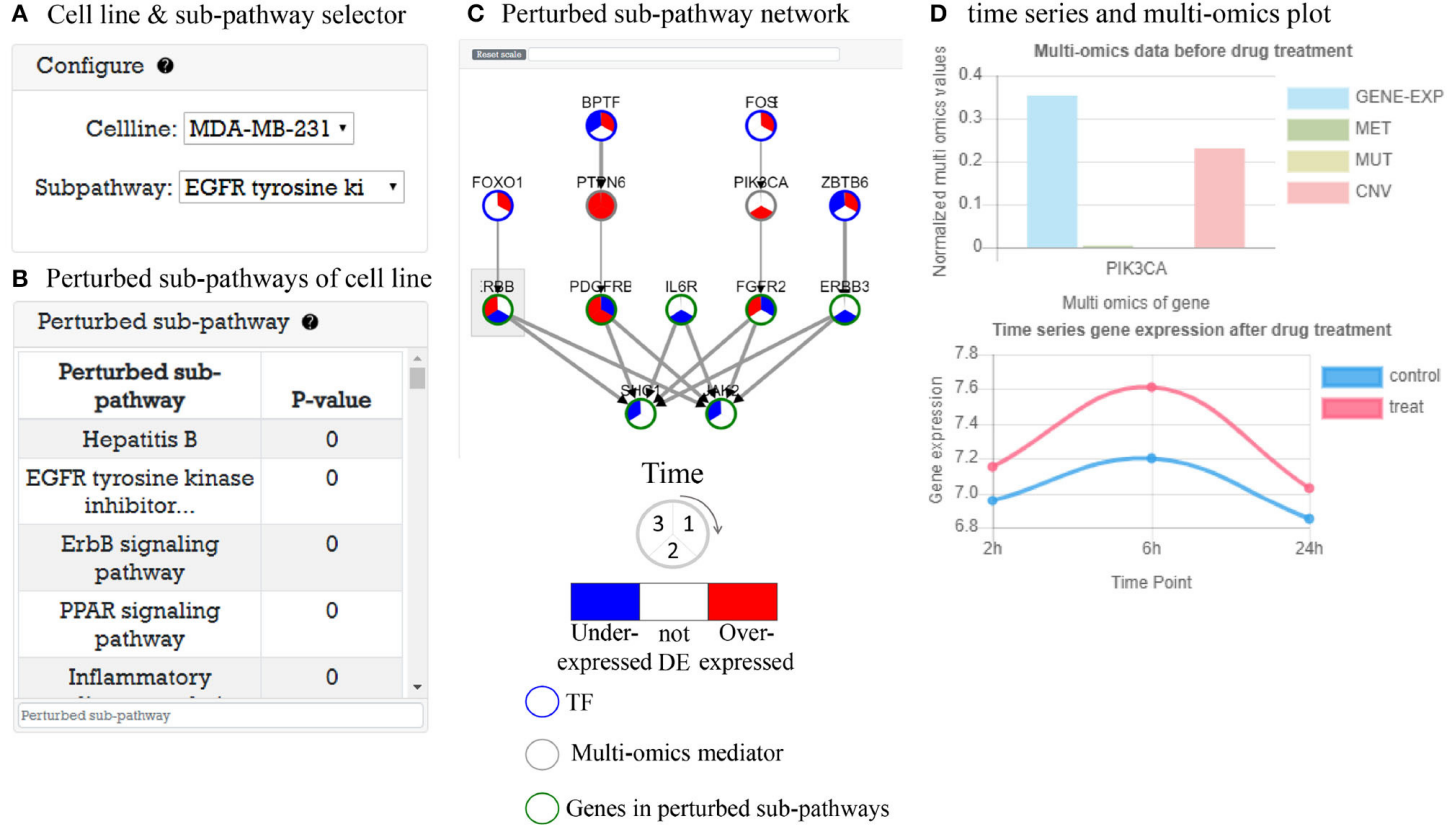
Time-series gene expression data analysis result after drug treatment. **(A)** Selector to visualize network of cell line. **(B)** A perturbed sub-pathway table of cell line. **(C)** Visualized time varying network TF to perturbed sub-pathway. **(D)** Gene information window that contains time-series gene expression plot and multi-omics data before drug treatment.

This part provides time-series gene expression data after drug treatment analysis results with perturbed sub-pathways. As an example, in Figure 5A, user can select cell line and perturbed sub-pathway to explore. When the user select a cell line, a perturbed sub-pathway table (Figure 5B) is generated with *P*-value. Figure 5C shows a TF-pathway network in time clock. When user clicks the gene node, a popup window appears to display multi-omics measurement of gene and expression plot of gene over time like Figure 5D. Furthermore, the user can search genes in the network. The user can control the network size by choosing a cut-off value for DEGs to identify perturbed pathway. If the cutoff is low, the number of nodes edges increases, which may cause false positive problems. In the opposite case, there may be a false negative problem. In either case, predicted perturbed pathways are computationally predicted, thus the user may need to further investigate perturbed pathways.

## 4. Case Study: Comparative Analysis of Breast Cancer Cell Lines That Have Different Sensitivity With Lapatinib

To demonstrate the usefulness of DRIM, we conducted an analysis on breast cancer cell lines in response to lapatinib administration. The lapatinib is a dual inhibitor on both targets epidermal growth factor receptor (EGFR) and human epidermal growth factor receptor 2 (HER2) tyrosine kinases (Medina and Goodin, 2008). It was approved by FDA in combination therapy for HER2-positive/overexpressed breast cancer patients. We chose five representative breast cancer cell lines that have distinct sensitivity/resistance on lapatinib (Table 2). These cell lines are all available on both multi-omics and time-series data to fully utilize the nature of DRIM.

**Table 2:** Five breast cancer cell lines that are available multi-omics data before drug treatment with lapatinib sensitivity and time-series gene expression data after drug treatment.

**Cell line**	**Molecular sub-subtype**	***IC*_50_(*μM*)**
BT-549	Basal B	2.02
T-47D	Luminal	2.90
MCF7	Luminal	3.04
MDA-MB-468	Basal A	3.77
MDA-MB-231	Basal B	6.50

### 4.1. Multi-Omics Analysis Result Before Drug Treatment

For multi-omics analysis for before drug treatment cells, DRIM selected *IC*_50_-related multi-omics potential mediator gene sets that are obtained by multi-omics integration analysis as shown in Figure 4. We carefully examined the set of candidate multi-omics potential mediator genes predictive of lapatinib sensitivity. The top 15 multi-omics potential mediator genes lapatinib are shown in Table 3 sorted by their relevance score with respect to lapatinib. Among the genes, ERBB3 (HER3) was previously known for its critical role in HER2-amplified breast cancer cells (Lee-Hoeflich et al., 2008). It is strongly associated with lapatinib sensitivity in coexpression with neuregulin-1 (NRG1) (Wilson et al., 2011). Genetic perturbations on other genes such as ABCG2, TP53, and HSF1 were also well known for lapatinib resistance (Rahko et al., 2003; Dai et al., 2008; Yallowitz et al., 2018).

**Table 3:** Top 15 multi-omics potential mediator genes that are related to lapatinib sensitivity.

**Genes**	**Score**
ERBB3	8.01
VEGFA	6.11
PGR	5.96
CDAN1	5.96
ABCG2	5.83
ESR1	5.71
CASP8	5.64
TP53	5.63
MAP2K7	5.62
CNTN4	5.53
DCTN6	5.44
CD274	5.39
NF2	5.31
CBL	5.19
E2F1	5.14

### 4.2. Time-Series Gene Expression Analysis Result After Drug Treatment

For the temporal pharmacogenomic analysis, we investigated cell line-specific perturbed sub-pathways that may be related to different lapatinib response. The lapatinib mainly targets PI3K signaling pathway, which plays a critical role in cell growth, survival, and proliferation (Fruman et al., 2017). Conceivably, aberrant activation of PI3K signaling is known to confer resistance to drugs targeting various receptor tyrosine kinases (Eichhorn et al., 2008; Wang et al., 2011). As expected, we collectively observed a significant time-course perturbation of PI3K signaling in each of the five cell lines in Table 4.

**Table 4:** The *P*-value of PI3K-Akt signaling pathway.

**Cell line**	***P*-value**
BT-549	1.6e-05
T-47D	6.07e-03
MCF7	7.11e-04
MDA-MB-468	4.94e-05
MDA-MB-231	1.08e-03

**Figure 6:**
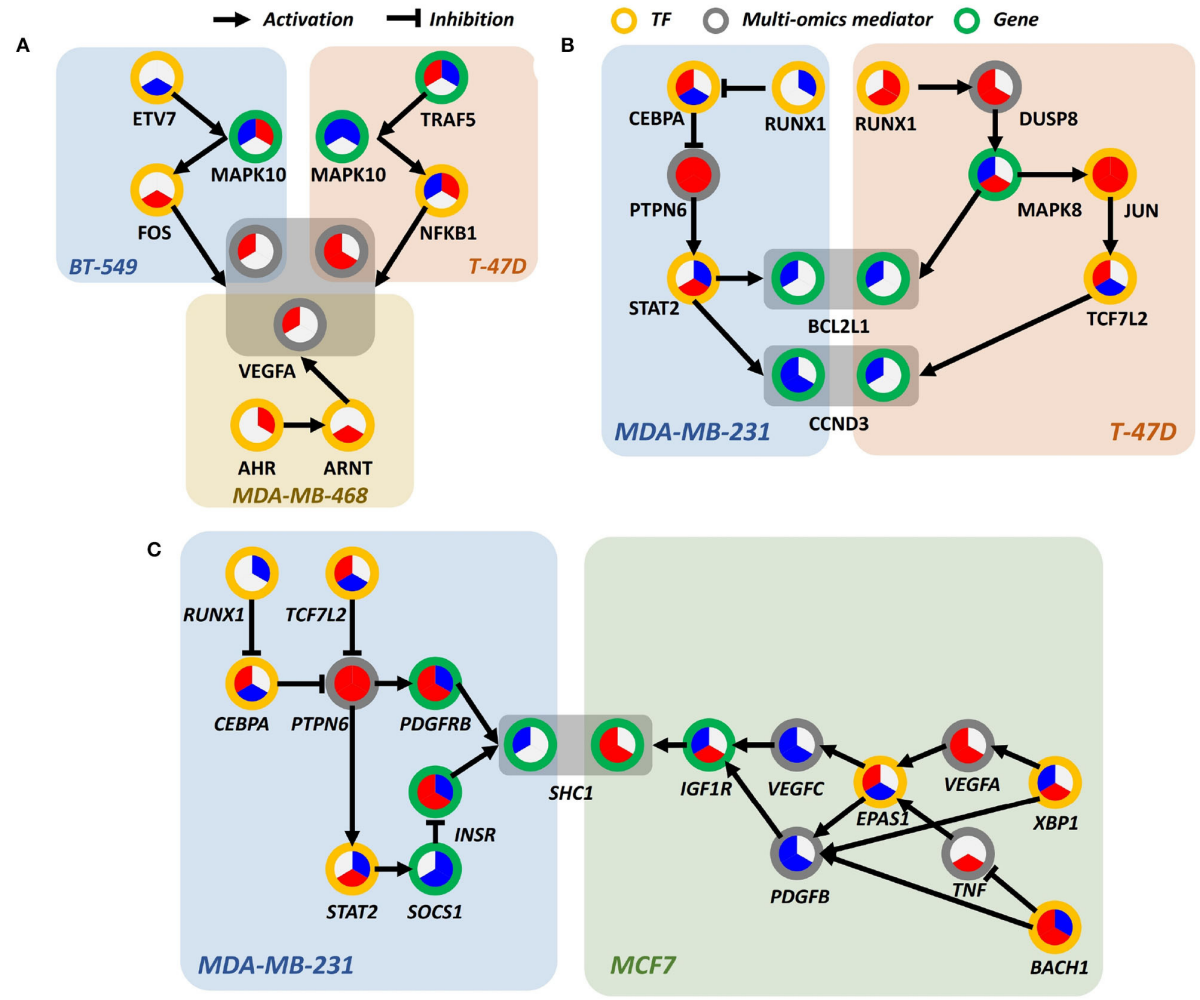
Differentially perturbed sub-pathway networks. **(A)** Regulatory path sharing VEGFA in BT-549, T-47D, and MDA-MB-468. **(B)** Regulatory path sharing CCND3, BCL2L1 in MDA-MB-231, T-47D. **(C)** Regulatory path sharing SHC1 in MDA-MB-231, MCF7.

We further examined in detail if there are differential sub-pathway level regulations among cell lines that mediate the response to the drug. Specifically, we asked whether each cell line harbors a distinct time-course regulatory path that governs the expression of a shared protein at the terminus of a pathway. To systematically identify such examples, we seeked for the regulatory paths with shared terminator protein for at least two cell lines using the “overview” network generated by DRIM. To simplify the analysis, we defined the terminator proteins as the nodes without outgoing edges in the network. Moreover, for biological interpretability, we only considered the paths starting from the transcription factors, and also enforced the paths to contain at least one multi-omics mediator. Different cell lines responded to lapatinib, accompanying distinct molecular perturbations, and shared the same terminal protein at the end of the paths (Figure 6).

Interestingly, we observed that many proteins involved in PI3K signaling pathway were regulated by different signaling pathways in a cell line-specific manner. For example, vascular endothelial growth factor A (VEGFA), a well-known effector molecule induced by PI3K signaling pathway (Karar and Maity, 2011), was shown to be activated through different signaling cascades, as shown in Figure 6A. In MDA-MB-468, VEGFA seemed to be induced by aryl hydrocarbon receptor (AhR) and aryl hydrocarbon receptor nuclear translocator (ARNT) signaling, presumably by the increased level of AhR/ARNT heterodimer as shown in Figure 6A. In BT-549 and T-47D cell lines, activation of JNK and NF-κB signaling was shown to be associated with increased level of VEGFA, respectively. Intriguingly, the time-bounded network allows the interpretation of the temporal difference of VEGFA induction between lapatinib-treated BT-549 and T-47D cell lines, as it can be deduced that the earlier response of T-47D was due to the more rapid induction of NF-κB than that of FOS in BT-549.

Bcl-2-like protein 1 (BCL2L1) and cyclin D3 (CCND3), overexpressed in human breast cancer, are anti-apoptotic proteins that delay cell death and increases cell survival (Simonian et al., 1997; Chi et al., 2015). In Figure 6B, in T-47D and MDA-MB-231 cell lines, BCL2L1 and CCND3 are downregulated in response to lapatinib that leads to cell death. In T-47D, overexpression of JUN throughout whole phases is a prominent characteristic. JUN is a well-known transcription regulator that induces apoptotic cell death (Bossy-Wetzel et al., 1997). It can be hypothesized that promoted cell death in response to lapatinib is attributed to the increased c-Jun.

Another interesting characteristic is that expression of downstream molecule of JUN—transcription factor 7-like 2 (TCF7L2)—increases over time, while T-47D cell line retained a high expression level of JUN. Since activity of c-Jun is predominantly regulated through phosphorylation, expression of molecules in regulatory relations should not be necessarily correlated. In MDA-MB-231, downregulation of BCL2L1 and CCND3 is induced by signal transducer and activator of transcription 2 (STAT2) (Furth, 2014), which involved in the JAK-STAT signaling pathway that leads to oncogenesis (Thomas et al., 2015). Although temporal relations between molecules are not clear, it still gives insight into which pathways are involved in elevated cell death.

SHC-transforming protein 1 (SHC1), a core regulator of receptor tyrosine kinase signaling, is an essential gene for promoting immune suppression. Downstream effects of SHC1 perturbation lead to STAT3/STAT1-related immune impairment. As previously mentioned, SHC1 can respond to EGF stimulation using multiple paths of protein phosphorylations and interactions (Zheng et al., 2013). There also exists PTPN12 as a turning point of SHC1 pTyr/Grb2 signaling that regulates cell invasion and morphogenesis. Reflecting the previous findings, our results on the perturbed sub-pathways also show multiple regulatory mechanisms that each of the breast cancer cell lines can potentially utilize favorable/possible sub-paths on the temporal flow, as shown in Figure 6C. This implies that upstream stimuli including EGF regulation direct multiple paths of temporal information among breast cancer cell lines (Zheng et al., 2013).

Even though all the five breast cancer cell lines were treated with the same drug, lapatinib, targeting receptors at cell surface with extreme specificity, each cell line showed different sensitivity to the drug. This heterogeneity may occur due to the complex crosstalk between various signaling pathways, which makes the inactivation of single signaling pathway by drug treatment not enough to cause systemic dysregulation of cellular machineries. Our system allows us to dissect this phenomena by differentially characterizing the fragments of regulatory cascades toward various effector molecules for each individual cell line as shown in Figure 6. Furthermore, since we have intracellular mechanistic portraits of drug response for each of the cell lines, it may allow us to devise novel combination therapeutic strategies targeting additional molecules that cells depend on after the primary drug is applied.

## 5. Discussion

For understanding the cell variability in drug response, personalized drug response analysis is demanded. In spite of increasing drug response genomics data, the interaction of high dimension multi-omics and time-series analysis are challenges for pharmacogenomics analysis. Pathway level analysis and multi-omics integration can be effective ways to interpret drug response data.

We developed an integrative multi-omics and time-series data analysis framework DRIM that finds perturbed sub-pathways and regulatory mechanisms in drug response. DRIM identifies the most likely regulatory path involving TF, multi-omics mediator gene, and perturbed sub-pathway for each cell line. DRIM provides analysis results in two perspectives. As a demonstration, we conducted an analysis of breast cancer cell lines that have different lapatinib sensitivity. In the multi-omics perspective result, DRIM selected multi-omics potential mediator genes that are related to lapatinib resistance in previous studies. In the temporal pharmacogenomic analysis result, we showed that DRIM can be used to discover distinct temporal regulatory mechanisms governing the induction of several common downstream proteins across cell lines.

## Data Availability Statement

CCLE multi-omics data with drug sensitivity analyzed for this study can be found in the depmap portal (https://depmap.org/portal/download/). NCI-60 cell line drug-treated time-series data analyzed for this study can be found in the GEO (https://www.ncbi.nlm.nih.gov/geo/query/acc.cgi?acc=GSE116451). LINCS-L1000 data analyzed for this study can be found in the GEO (https://www.ncbi.nlm.nih.gov/geo/query/acc.cgi?acc=GSE92742).

## Author Contributions

SK designed the project. SK, MO, SLe, and SLi designed drug response analysis algorithm. MO and IJ implemented multi-omics integration algorithm. MO, SP, and KJ implemented time-series analysis algorithm. MO and SP implemented web system. DJ designed workflow and web system. SK, SLe, SLi, DL, and DJ biologically interpreted the drug response analysis results. SK, MO, SLe, SLi, DL, DJ, and SP wrote and revised the paper. All authors contributed to the article and approved the submitted version.

## Conflict of Interest

The authors declare that the research was conducted in the absence of any commercial or financial relationships that could be construed as a potential conflict of interest.
